# Using Undergraduate Researchers to Build Vector and West Nile Virus Surveillance Capacity

**DOI:** 10.3390/ijerph10083192

**Published:** 2013-07-31

**Authors:** Grant Hokit, Sam Alvey, Jennifer M. O. Geiger, Gregory D. Johnson, Marni G. Rolston, Daniel T. Kinsey, Neva Tall Bear

**Affiliations:** 1 Department of Natural Science, Carroll College, 1601 N. Benton Ave., Helena, MT 59625, USA; E-Mails: salvey@carroll.edu (S.A.); jgeiger@carroll.edu (J.M.O.G.); 2 Montana State University, Bozeman, MT 59717, USA; E-Mails: gdj@montana.edu (G.D.J.); mrolston@montana.edu (M.G.R.); 3 Aaniiih Nakoda College, Harlem, MT 59526, USA; E-Mail: dkinsey@ancollege.edu; 4 Little Big Horn College, Crow Agency, MT 59022, USA; E-Mail: tallbearn@lbhc.edu

**Keywords:** West Nile Virus, vector surveillance, *Culex tarsalis*, arthropod vectors, infectious disease

## Abstract

Vector surveillance for infectious diseases is labor intensive and constantly threatened by budget decisions. We report on outcomes of an undergraduate research experience designed to build surveillance capacity for West Nile Virus (WNV) in Montana (USA). Students maintained weekly trapping stations for mosquitoes and implemented assays to test for WNV in pools of *Culex tarsalis*. Test results were verified in a partnership with the state health laboratory and disseminated to the ArboNET Surveillance System. Combined with prior surveillance data, *Cx. tarsalis* accounted for 12% of mosquitoes with a mean capture rate of 74 (±SD = 118) *Cx. tarsalis* females per trap and a minimum infection rate of 0.3 infected mosquitoes per 1000 individuals. However, capture and infection rates varied greatly across years and locations. Infection rate, but not capture rate, was positively associated with the number of WNV human cases (Spearman’s rho = 0.94, *p* < 0.001). In most years, detection of the first positive mosquito pool occurred at least a week prior to the first reported human case. We suggest that undergraduate research can increase vector surveillance capacity while providing effective learning opportunities for students.

## 1. Introduction

Infectious disease is a persistent public health concern. In addition to traditional threats, 36 newly emerging infectious diseases have been described within the past four decades and the majority of recent outbreaks involve vector-borne zoonotic diseases [1,2,3]. Vector surveillance provides early detection of potential outbreaks leading to accurate application of vector control, targeted public awareness, and better allocation of medical resources [2,3,4,5,6]. Although the cost effectiveness of surveillance programs has been repeatedly demonstrated (e.g., [6,7,8]), government funded surveillance programs are continually threatened by budget concerns [1,2,5].

Passive surveillance of health-care reporting is less costly and has been proposed as an alternative to active vector surveillance [2,4]. However, passive surveillance relies on voluntary reports of symptoms or diagnoses by health care institutions. Variability in reporting efforts, poor geographic precision, and analysis of post-infection data are significant challenges faced by passive surveillance programs. Although passive surveillance does not preclude vector control, without data on the location and phenology of vectors, control measures may be less effective. The lack or ineffectiveness of vector control may facilitate outbreaks as suggested by the global resurgence of yellow fever [9], the West Nile virus outbreak in New York [10], and recent cases of dengue in Florida and Texas [11]. The investment in vector surveillance coupled with vector control can save up to 10 times the economic cost compared to a program that relies on post-infection controls [8]. 

Although already cost effective, initial investments in vector surveillance may be further reduced through the efforts of undergraduate student researchers. Recent developments in higher education have emphasized the value of apprentice-based learning and the development of undergraduate research programs [12]. Providing undergraduate students with authentic research experiences leads to better understanding of the scientific process, competency in scientific techniques, longer retention of scientific information, and better preparation for advanced studies [13,14] and the effects appear stronger for minority students who are underrepresented in the sciences [15]. Using undergraduates for vector surveillance provides research experience for students and can reduce costs associated with surveillance programs.

We report on the implementation and results of a vector surveillance program designed to detect the presence of West Nile virus (WNV) in the state of Montana. First reported in the United States in New York in 1999, WNV has spread quickly and was documented in Montana in 2002 [16]. Using undergraduate students from five collaborative institutions, we have implemented a program that samples known mosquito vectors from across the state, shares samples with the state public health laboratory, tests for WNV in competent vectors, and reports results to county, state and federal health officials. 

## 2. Experimental Section

### 2.1. Student Recruitment and Training

Four undergraduate institutions (Aaniiih Nakoda College, Carroll College, Chief Dull Knife College, and Little Big Horn College) and one university (Montana State University) participated in our vector surveillance program. Participation of all five institutions enabled broad geographic coverage of the state of Montana that has an area of 380,849 km^2^ and provided research opportunities to students at relatively under-resourced tribal colleges. 

Preparation for each trapping season began in the winter months when each undergraduate institution recruited at least two students (more if funds allowed) to participate in a summer undergraduate research program. We required students to attend an orientation meeting for student researchers, submit a student research application, and complete an interview with one of the faculty mentors located at each institution. Criteria for selection of a student included academic merit, course work completed, availability during the summer season, and reasons for pursuing undergraduate research. Those students interested in a research career were given priority.

In spring or early summer, we required student researchers to participate in two training workshops. Mosquito Identification and Ecology hosted by Montana State University provided students with an introduction to mosquito life history, species identification, species habitat associations, trapping protocols, collection handling and sorting, and details concerning WNV amplification in competent vectors. Molecular Detection Protocols hosted by Carroll College provided training on sample preparation, RNA extraction techniques, and WNV detection protocols.

### 2.2. Sampling Protocol

Surveillance methods were designed to be in compliance with the Center for Disease Control (CDC) guidelines for WNV surveillance [17] and included both “fixed” and “flexible” trapping stations. Students at undergraduate institutions each maintained 3–5 fixed stations where mosquitoes were trapped most weeks from mid-June through August from 2009–2012. We located fixed stations in areas with known or suspected WNV activity. We included flexible stations, where trapping occurred in some weeks and/or some years, during peak season (late July through early August) when funding opportunities allowed for more students, travel, and supplies. Collections from other flexible sites were mailed to Carroll College from county extension agencies with properly equipped mosquito control personnel. Flexible stations increased the geographic distribution of collections.

Students collected mosquitoes using CDC miniature light traps (J.W. Hock, Gainesville, FL, USA) baited with CO_2_ either from a compressed gas tank or dry ice. Traps operated from 1–2 h before dusk to 1–2 h after dawn. Mosquito collections were transported to the laboratory in coolers and frozen at 20 °C for ≥24 h to ensure mosquito mortality before processing. Students processed collections ≤1,000 mosquitoes by examining all individuals on a chill table under a stereo microscope and sorting *Culex tarsalis*, the main vector for WNV in Montana [16], into pools of ≤50 female mosquitoes. Sorting continued for collections ≥1,000 mosquitoes up to 3,000 individuals only if *Cx. tarsalis* was observed in the first 1,000 individuals. Students stored pools of *Cx. tarsalis* in 1.5 mL screw cap vials containing a ceramic bead and RNALater (Qiagen Inc., Valencia, CA, USA), at −80 °C until analysis.

### 2.3. Detection Protocol

We homogenized pools containing ≤10 mosquitoes in 600 mL of RNALater and 300 mL of BA-1 homogenate buffer [18], while pools ≥ 10 mosquitoes received 1,000 mL and 500 mL, respectively. Tubes were placed in a 115V FastPrep FP120 (Thermo Fisher Scientific Inc., Waltham, MA, USA) and homogenized for 30 seconds at a speed of 5 m/s. Extraction of RNA was completed as directed by the QIAamp Fibrous Tissue RNA Kit (Qiagen Inc.). RNA extracted samples were stored at −80 °C until testing.

To test for the presence of WNV in mosquito pools, we followed well-documented protocols [18] and used 2 sets of primers and probes (WNENV and WN3’NC) in a TaqMan RT-PCR. Positive controls were established by extracting RNA from 100 mL of NATtrol WNV (ZeptoMetrix Corporation, Buffalo, NY, USA) using the QIAamp Viral RNA Mini Kit (Qiagen Inc.). Water was used as a negative control. The TaqMan RT-PCR was performed with the iQ5 Real-Time PCR Detection System (Bio-Rad Laboratories, Hercules, CA, USA) using a total reaction volume of 50 µL containing 5 µL RNA extract, 1 µL of 50 pmol/mL forward and reverse primer, 2 µL of 10 pmol/µL probe, 1.25 µL of MultiScribe Reverse Transcriptase (Applied Biosystems Inc., Foster City, CA, USA), and 25 µL of TaqMan Universal PCR Master Mix (Applied Biosystems Inc.) [19]. The final volume was brought to 50 µL with water. The thermocycling conditions used were 30 min at 48 °C, 10 min at 95 °C, and 55 cycles of 15 seconds at 95 °C and 1 min at 60 °C.

We analyzed each sample in duplicate for each of the two primer/probe sets to rule out cross contamination as a source of false positives. Samples with a cycle threshold (Ct) value <35.0 were considered positive. Samples with a Ct value ≥35.0 were considered tentative-positives pending further testing. Samples were considered negative if both duplicates failed to reach the threshold value. If the Ct value was greater than 35, or if only one of the duplicates tested positive, the sample was run a second time to validate results. Thus, we implemented extensive methods to minimize the possibility of erroneous results, including, (1) the presence of both positive and negative controls in each test, (2) the use of 2 separate probes in each test, (3) duplicating each sample in each test, and (4) repeating the entire procedure for any result with a Ct value greater than 35. Results were confirmed positive only when all 8 results (2 probes by 2 samples by 2 tests) were in agreement. As a further control against spurious results, from 2010–2012, homogenates from all samples were shared and tested in parallel at the Montana Public Health Laboratory of the Montana Department of Public Health and Human Services (DPHHS). 

### 2.4. Data Analysis and Dissemination

To provide an estimate of capture rate for *Cx. tarsalis*, we calculated a mean light trap index (LTI = number of individual *Cx. tarsalis* per trap) [20,21,22] in aggregate (across all sites for all years) and by individual site per year. A corresponding mosquito infection rate was estimated to compare *Cx. tarsalis* abundance with infection risk. For simple aggregate comparisons, we estimated minimum infection rate (MIR) [23] as the number of positive pools divided by the number of *Cx. tarsalis* tested multiplied by 1,000 to estimate the minimum number of females infected per 1,000 mosquitoes. We used Spearman’s rank correlation analysis to test for associations between LTI and MIR and to test for associations between each of these parameters and the number of human cases reported in Montana. Human case data were obtained from the CDC ArboNet Surveillance System.

Sample pools were considered positive only by cross-validation at our laboratory and DPHHS. Positive results were reported to the CDC ArboNET Surveillance System within 7–10 days of testing. In compliance with the CDC guidelines for WNV surveillance [17], the ArboNET report included the date samples were collected, state, county, mosquito species, number of mosquitoes collected, number of mosquitoes tested, and a unique identifier for each positive pool. Additionally, annual reports were provided to the Montana chapter of the Northwest Mosquito and Vector Control Association.

### 2.5. Assessing Student Outcomes

We assessed the educational value of the program using the following metrics: number of student participants, number of students matriculating into advanced science/health degrees, and the number of student-authored scientific products (e.g., presentations and publications).

## 3. Results and Discussion

### 3.1. Surveillance Results

**Figure 1:**
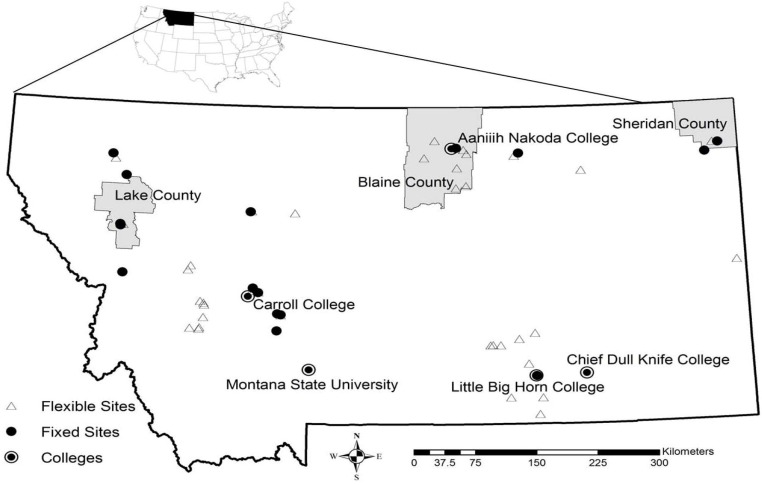
Fixed and flexible surveillance locations for 2009–2012. Fixed sites were trapped ≥10 times during the 10-week period from the third week in June through August. Flexible sites were trapped fewer than 10 times or only in some years as funding allowed.

Fifteen fixed stations were trapped ≥10 times during the 10-week trapping season from 2009–2012 and were augmented with flexible sites trapped ≤10 times (Figure 1). When combined with 77 sites from previous studies, (e.g., [16]) there are now 129 georeferenced capture sites for *Cx. tarsalis* in Montana.

Between 2009–2012 students collected an estimated 1,026,912 mosquitoes over 810 trap nights, examined 492,139 individual mosquitoes, and sorted 59,956 *Cx. tarsalis* into 1,202 pools for WNV testing of which 17 were confirmed positive by both our laboratory and DPHHS. Thus, overall *Cx. tarsalis* composed 12% of the examined mosquitoes; the mean capture rate was 74 (±SD 118) *Cx. tarsalis* per trap night; and the MIR over 4 years was 0.3 females infected per 1,000 mosquitoes. However, as Table 1 indicates, mosquito abundance (LTI range = 23–181) and mosquito infection rate (MIR range = 0.0–1.7) varied depending on the year. This variation was even greater when student data were combined with data from previous years that used identical sampling protocol provided by Johnson ([16], unpublished), with MIR ranging from 0.0–14.9. This variability was reflected in the annual number of human cases for Montana obtained from ArboNET (Table 1). By comparing the date of first detection in a mosquito pool with the week of the first reported human case in ArboNet, we observed that in most years the virus was detected in mosquitoes in Montana before the first human case. In 5 of 9 years, positive mosquito pools were detected 1–4 weeks before the first human case; in 3 of 9 years, mosquito pools were found positive in the same week as the first human case; and in only 1 of 9 years a human case was reported 1 week before a mosquito pool tested positive.

**Table 1:** Mosquito collection results aggregated across all sites for each year. The mean light trap index provides a measure of *Cx. tarsalis* abundance across years standardized by trapping effort. The minimum infection rate provides an estimate of the number of infected females per 1000 mosquitoes. Our data (2009–2010) is compared with data from previous years provided by Johnson *et al*. ([16], unpublished). Human data were obtained from ArboNET. LTI = light trap index = number of sorted *Cx. tarsalis* divided by the number of traps; and MIR = minimum infection rate = number of positive samples divided by the number of *Cx. tarsalis* tested multiplied by 1,000.

Year	No.	*Cx. tarsalis*	No. *Cx. tarsalis*	No.	MIR	Human
	trap nights	mean LTI	pools tested	positive		cases
2003	180	50	389	134	14.9	228
2004	380	38	323	6	1.3	7
2005	289	67	557	15	0.8	25
2006	261	50	326	29	2.2	34
2007	222	95	458	78	3.7	202
2008	94	46	118	3	0.7	5
2009	204	35	145	5	0.7	5
2010	201	23	92	1	0.2	0
2011	230	181	832	0	0.0	1
2012	175	38	133	11	1.7	6

In addition to annual fluctuations, mosquito surveillance results varied geographically across the state. For example, combined with data from previous years, student data provided an 8 year summary of *Cx. tarsalis* mean LTI and MIR for 3 counties from across the state along an east-west transect: Sheridan County in northeastern Montana, Blaine County in north-central Montana and Lake County in western Montana (Table 2). The mean LTI varied across years for all 3 sites but was consistently higher in Sheridan and Blaine counties compared to Lake County. Likewise, MIR varied annually for Sheridan and Blaine counties but no positive pools were confirmed in Lake County for *Cx. tarsalis* (2 positive pools were confirmed for *Cx. pipiens* in Lake County in 2007).

**Table 2:** Mean light trap index (LTI = number of sorted *Cx. tarsalis* divided by the number of traps) and minimum infection rate (number of positive samples divided by the number of *Cx. tarsalis* tested multiplied by 1,000) for 3 counties from different parts of Montana. Our data (2009–2010) is compared with data from previous years provided by Johnson *et al*. ([16], unpublished).

Year	Sheridan County		Blaine County		Lake County	
	LTI	MIR	LTI	MIR	LTI	MIR
2005	210.4	1.4	86.0	0.0	39.8	0.0
2006	131.0	1.3	245.0	3.6	27.3	0.0
2007	334.0	6.0	185.0	7.6	44.7	0.0
2008	62.6	3.2	113.3	1.1	50.1	0.0
2009	197.5	1.0	152.7	0.7	25.8	0.0
2010	380.0	1.1	75.1	0.0	37.0	0.0
2011	1,690.0	0.0	204.6	0.0	46.9	0.0
2012	21.6	5.8	80.8	6.2	21.6	0.0

Our results suggest that mosquito infection rates vary but are consistently higher in north-central and eastern Montana compared to western Montana. This reflects human case data that show 6–7 times more human cases *per capita* in eastern Montana counties [24]. On a larger scale, although WNV has now been reported in every continental U.S. state and most Canadian provinces, the distribution of WNV across North America is not homogenous. Several geographic, climatic and biological factors have been associated with the distribution of WNV and the resulting heterogeneous distribution suggests that infection risk is not uniform [25,26,27,28]. For the purposes of an early-warning system in Montana, it may be important to bias trapping density east of the Continental Divide.

### 3.2. Patterns in Infection Rate

Although highly variable, the abundance (as measured by LTI) of *Cx. tarsalis* was not associated with mosquito infection rates (as measured by MIR) across years (Spearman’s rho = 0.23, *p* = 0.519). Also, LTI was not associated with the number of human cases across years (Spearman’s rho = 0.43, *p* = 0.210). However, the human cases were higher in years with larger MIR values (Spearman’s rho = 0.94, *p* < 0.001). These results suggest that infection risk is not associated with yearly fluctuations in abundance of the mosquito vector. The mosquito infection rate and the number of human cases varied independent of *Cx. tarsalis* capture numbers. 

The absence of a correlation between vector abundance and infection rates has been observed in other studies [26,29,30] although the explanation is often speculative. A decoupling between vector abundance and infection may occur through ecological and/or physiological mechanisms that influence pathogen-vector-host relationships. For example, the decoupling may result from modified host behavior in the presence of large numbers of mosquitoes such as humans sleeping under mosquito nets [30], birds dispersing/migrating from infested areas [29], or other mosquito defense behaviors [31]. Alternatively, host abundance, immunity, diversity and distribution may influence vector infection rates (e.g., [28,32]). Also, as has been observed for WNV, the pathogen may directly affect the abundance of the vector by decreasing survivorship and/or fecundity [33,34]. Even the age-structure of a mosquito population may influence infection rates [35]. Finally, environmental thresholds may influence infection rates, as when the minimum temperature for development is lower for mosquitoes than for WNV [36] resulting in *Cx. tarsalis* populations in locations where WNV is not possible or improbable [25]. In fact, local temperature is often cited as an important parameter in predicting vector infection and human cases for WNV [37,38]. Even large, daily temperature fluctuations, without an associated change in the overall mean temperature, can inhibit the development of a pathogen [39]. Regardless of the mechanism, vector abundance does not always predict infection rates or human cases. Consequently, vector surveillance should be coupled with a pathogen detection protocol. 

### 3.3. Student Outcomes

A total of 39 students participated in the project between 2009–2012. Student outcomes include: three students matriculating from 2-year tribal colleges into science/health programs at larger universities; two students matriculating into Post-Baccalaureate Fellowships at the National Institute of Health; and six, three and one students matriculating into graduate research programs, medical schools, and pharmacy school, respectively. Also included are 22 student-authored presentations at scientific conferences and three student-authored manuscripts submitted for publication. Several other faculty-student collaborations are ongoing. 

Our results demonstrate the effectiveness of using undergraduate researchers to implement a vector surveillance program. At a cost of approximately $150,000 per year to train, equip and compensate student researchers and faculty mentors, we implemented a vector surveillance program that provided public health officials and mosquito control agencies with information regarding WNV activity in Montana. In some years student samples were the only samples provided to the state health laboratory in Montana. At an estimated $40,000 per human case [40] for post-infection, health-care costs (not to mention death and suffering), the value of a vector surveillance program is apparent even for a state like Montana that reports few human cases in most years.

## 4. Conclusions

Our results combined with data from ArboNET suggest that, from year to year, mosquito infection rates are associated with the number of WNV human cases in Montana and that detection of the first positive mosquito pool occurs prior to the first reported human case. Thus, frequent testing for WNV in vector pools can be useful for predicting human susceptibility. Active vector surveillance requires field and laboratory technicians to maintain trapping stations, sort captured arthropods, test for target pathogens and report results to a centralized database. The human resources costs make vector surveillance particularly vulnerable to budget cuts. Although an investment is required, vector surveillance allows for preemptive control measures applied with geographic precision. Furthermore, additional vector surveillance data may provide a better understanding of the causes and consequences of WNV distribution patterns resulting in more accurate predictions of WNV epidemics and more effective preventative controls (e.g., mosquito control and public health warnings). 
